# Homelessness and housing problems in admitted psychiatric patients in Flanders, Belgium

**DOI:** 10.3389/fpubh.2024.1392558

**Published:** 2024-06-21

**Authors:** Kirsten Catthoor, Kris Van den Broeck, Mathilde Hage, Luna Van Suetendael, Yves Wuyts, Geert Van Isterdael, Marc De Hert

**Affiliations:** ^1^Working Group “Poor makes sick, sick makes poor” of the Estates-General of Mental Health, Kortenberg, Belgium; ^2^Flemish Association of Psychiatry, Kortenberg, Belgium; ^3^Collaborative Antwerp Psychiatric Research Institute (CAPRI), University of Antwerp, Antwerp, Belgium; ^4^Ziekenhuis Netwerk Antwerpen, Psychiatric Hospital Stuivenberg, Antwerp, Belgium; ^5^Family Medicine & Population Health (FAMPOP), University of Antwerp, Antwerp, Belgium; ^6^London School of Economics Law School, The London School of Economics and Political Science, London, United Kingdom; ^7^Zorgnet-Icuro, Brussels, Belgium; ^8^UilenSpiegel, Brussels, Belgium; ^9^University Psychiatric Center Katholieke Universiteit Leuven, Kortenberg, Belgium; ^10^Department of Neurosciences, Center for Clinical Psychiatry, Katholieke Universiteit Leuven, Leuven, Belgium; ^11^Leuven Brain Institute, Katholieke Universiteit Leuven, Leuven, Belgium; ^12^Antwerp Health Law and Ethics Chair, University of Antwerp, Antwerp, Belgium

**Keywords:** homelessness, psychiatric patients, psychiatric hospitals, psychiatric wards in general hospitals, Flanders, Belgium, social service, action plan

## Abstract

Homelessness in psychiatric patients in Flanders, Belgium, has never been investigated. Advocacy groups from patients with lived experience of psychiatric disorders have sounded the alarm on the scarcity of suitable housing options, the strain on psychiatric institutions, and the challenges faced by social service workers. To investigate the extent of the problem a survey on the topic was initiated. A “homelessness-in-mental-health-questionnaire” was designed by experts in the field. The social services of all Flemish psychiatric hospitals and all psychiatric wards in general hospitals were contacted and invited to complete this survey. 24 of 70 contacted services responded. The total number of homeless patients in the inpatient setting on an annual basis are estimated to an average 19.5%. 18% of homeless patients remain longer in admission due to the lack of housing options. 13.7% of homeless psychiatric patients are referred to a community care facility such as an assisted living facility. Social service respondents reported spending an average of 27.4% of their work time on housing issues. The main focus points according to the respondents are the lack of priority measures for homeless psychiatric patients, psychiatric problems as a barrier to housing options and the shortage of adapted housing capacity. The conclusion of this study is the need for comprehensive policy interventions to ensure an adequate supply of suitable social housing for psychiatric patients, accessible mental health care, alternative housing options and crisis accommodation facilities. We propose a 10-point action plan on housing for psychiatric patients for policymakers and politicians.

## 1 Introduction

Extra-clinical factors such as the unavailability of suitable housing options are important determinants of prolonged hospitalization in acute inpatient settings (1). Many patients who exhibit a revolving door pattern of multiple hospitalizations are people living homeless (2). Homelessness and problems of residence remain a challenge for many patients with severe mental illness, and community mental health services are still far away from providing adequate treatment to this population (2).

Houselessness and homelessness cause far-reaching negative effects on physical and mental health. Research has shown increased incidence of malnutrition, chronic pain, skin diseases, musculoskeletal disorders, poor dental health, respiratory disorders such as community-acquired pneumonia, asthma and chronic obstructive pulmonary disease and infectious diseases such as tuberculosis, hepatitis C virus, Human Immunodeficiency Virus (HIV) and Acquired Immunodeficiency Syndrome (AIDS) in psychiatric patients facing homelessness (3). A systematic review on the prevalence of mental disorders and major psychiatric diagnoses in homeless populations in high-income countries estimated any current mental disorder at 76.2%, with alcohol use disorders at 36.7%, drug use disorders at 21.7%, schizophrenia spectrum disorders at 12.4% and major depression at 12.6% (4). Accordingly, mortality rates are estimated to be fourfold (4). A systematic review and meta-analysis from 2024 showed comparable but slightly lower results (5). Men showed a significantly higher lifetime prevalence of mental health disorders (86%) compared to women (69%). The prevalence of specific disorders was 44% for any substance use disorder, 26% for antisocial personality disorder, 19% for major depression, 7% for schizophrenia, and 8% for bipolar disorder (5). This starkly contrasts with figures on psychiatric disorders in the general population. According to the most recent Global Burden of Disease study in 2019 (6), in high-income countries, the age standardized prevalence of schizophrenia and depressive disorders are estimated at 0.3% and 3.7%, respectively.

Research also shows that, whenever homeless people get into the hospital, the costs of treatment are higher compared to those of non-homeless patients (7). The elevated costs are partly explained by the length of stay, which is generally prolonged due to the mental health problems these patients have (7). Yet, an important share of the higher costs could not be explained by a prolonged length of stay alone (8). Disease severity at admission reflecting the limited availability of community mental health services is most probably the main reason of higher financial costs (8).

All major independent international organizations agree that policy decisions on healthy living should be a short-term priority. The World Health Organization states that improved housing conditions can save lives, prevent disease, increase quality of life, reduce poverty, help mitigate climate change and contribute to the achievement of the Sustainable Development Goals, including those addressing health and sustainable cities (9) The World Economic Forum advises non-profit organizations to accept the critical role in bridging the gap between governments and the private sector to improve the affordability of housing, as well as working with individuals to help them understand their options and make informed decisions (10).

Policy recommendations on the health of homeless people in high income countries (11) not only mentioned the need for establishment of homeless teams in all metropolitan centers, but also emphasizes the importance of clarifying the costs of such services. Recent research on homelessness patients in an inpatient psychiatric care settings in Berlin showed an 15.1% increase rate on a 13-year period (2008–2021) (12). On the socio-demographical level of this study findings, remarkable conclusions were the lack of expanding social housing capacity despite a substantial growing population, and a comparable disequilibrium between the rising number of inhabitants and a much slower increasing inpatient mental health care (12).

Although homeless patients with severe psychiatric disorders pose a significant challenge for healthcare and social support services, positive outcomes have been reported with various forms of treatment. A systematic review on psychosocial interventions for homeless individuals with mental illness demonstrates the positive effects of critical time intervention, case management, housing support intervention, assertive community treatment, and life skills training on sustaining housing stability, preventing relapse, reducing hospitalizations, and improving quality of life of the homeless persons with mental illness (13). A systematic review and meta-analysis on housing first (homeless assistance approach that prioritizes providing permanent housing to people experiencing homelessness without prerequisites), shows not only a reduction on homelessness and non-routine health service use, but also a limitation in problematic substance use (14).

Advocacy groups from patients with lived experience of psychiatric problems in Belgium have sounded the alarm on the severity of housing problems in the psychiatric population in Belgium, the number of homeless patients in psychiatric wards and its impact on mental health treatment in the task force group “Poor makes sick, sick makes poor” from the Estates General of Mental Health (EGMH) (15). The task force group consists of a delegation of mental health professionals, including a psychiatrist, psychologist, pharmacist, policy maker and patients with lived experience of psychiatric problems. The EGMH is an organization of all interested stakeholders within the extended mental health sector (16). The EGMH aspires to arrive at a shared vision of the current strengths and vulnerabilities within the mental health sector and translate them into policy recommendations and priorities for change (16).

Persons with lived experience of psychiatric illness in the “Poor makes sick, sick makes poor” task force group indicated that there are significant problems in terms of housing and sustainable residence for individuals with mental health problems (15). There is a fundamental shortage of affordable housing, leading to significant challenges for many psychiatric patients. Some of them become homeless, and there are no alternative housing options available. They end up in dire conditions in psychiatric institutions, where there is also no solution for their housing problems. Hypothesis are made that the large number of patients with residential problems admitted to psychiatric institutions are partly the cause of long waiting times for admission for other patients. In addition to the delay factor for admission of other acutely ill patients, homeless patients are thought to require so much time investment from therapeutic staff, especially social services, that regular care is compromised. Yet, there are no studies assessing the actual time investment of therapeutic staff.

A study in Belgium in 2022 demonstrated that thousands of deaths could be prevented if all neighborhoods had the same low mortality rates as the least deprived areas regarding housing conditions (17). Identifying and addressing hotspots of housing inequality with specific public interventions is crucial (17). On the other hand, there is no information available about the number of homeless individuals in Belgium, nor about the number among them who have psychiatric issues. The interdisciplinary knowledge center LUCAS at the Catholic University of Leuven is currently assessing local counts of individuals experiencing homelessness. The results of these counts from 3 major cities (Antwerp, Ghent and Leuven) are now available, but with substantial missing values (18). During the observation period of this study, 46% of homeless persons temporarily admitted to an institution in Antwerp, were staying in a psychiatric hospital or a psychiatric ward in a general hospital (19).

The task force group “Poor makes sick, sick makes poor” decided to initiate a survey on the extent of the housing crisis in psychiatric patients in Flanders, by exploring the proportion of homeless patients in psychiatric hospitals and psychiatric wards in general hospitals. Besides, its impact on the workload of social workers in psychiatric institutions was to be evaluated. The purpose of the research was to gather sufficient data and substantive material that can be used in negotiations with policymakers and the government, in order to strengthen and improve the position of psychiatric patients.

## 2 Methods

In April 2023, 4 members of the task force group “Poor makes sick, sick makes poor” (psychiatrist, psychologist, policymaker, and person with living experience of psychiatric disorder) developed a 15-item questionnaire on the issue of homelessness within the walls of a psychiatric hospital or a psychiatric ward in a general hospital, from the perspective of the social worker (Table 1). The position of the social worker was chosen as the focus because from the professional point of view, as they are most directly confronted with the issue of homelessness. The questionnaire consists of 15 questions, all directly addressing the issue of homelessness in psychiatric hospitals or psychiatric wards within general hospitals. Some of the questions involve estimates of the proportion of homeless patients overall, and the extended stay in the facility due to homelessness. This part is considered as the quantitative part of the enquiry. The other part of the questionnaire is more qualitative in nature, such as description of priority collaborations with social housing agencies.

**Table 1:** 15-item questionnaire on the problem of homelessness from the social service point of view.

What percentage of patients admitted on an annual basis are confronted with homelessness?	0–100%**	
What percentage of all present patients stay longer in the hospital solely due to housing issues?	0–100%**	
What percentage of homeless psychiatric patients are referred to another residential facility upon discharge, while they might be capable of independent living if adequately supported housing were available?	0–100%**	Assisted living facility for the older adults or a psychiatric nursing home
What proportion of homeless patients utilize prioritized allocation in social housing?	0–100%**	
What percentage of patients utilizing prioritized allocation in social housing are obliged to accept certain forms of care/support/follow-up?	0–100%**	
Does your hospital have a protocol/procedure/established method regarding the issue of homeless patients?	0 or 100**	n/y
Does your hospital implement an active policy regarding the prevention of evictions?	0 or 100**	n/y
What percentage of potentially homeless patients is this active policy applied to?	0–100%**	
What priority collaborations does your hospital have with other partners regarding finding housing for homeless patients?		OCMW*, emergency housing, social housing companies, social rental agencies, real estate agencies, abbeys or monasteries, hotels, homeless shelters run by CAW*, municipal or city-owned houses, hotels.
What percentage of the workload of the social service is spent on assisting patients with their housing issues?	0–100%**	
How would you rate the average housing provided to a patient with mental health issues by a social housing organization?		On a scale of 0 (completely unsuitable) to 10 (fully suitable)
How much insight does a housing landlord have into the specific vulnerabilities or needs of individuals with mental health vulnerabilities?		On a scale of 0 (completely unsuitable) to 10 (fully suitable)
How much insight do housing policymakers have into the specific vulnerabilities or needs of individuals with mental health vulnerabilities?		On a scale of 0 (completely unsuitable) to 10 (fully suitable)
According to you, what are the necessary steps to improve policies regarding housing issues for psychiatric patients?		Open-ended question
According to you, what are features that makes a living space suitable for psychiatric patients?		Open-ended question

*OCMW, openbaar centrum voor maatschappelijk welzijn (public center for social welfare); CAW, Centrum voor Algemeen Welzijn (Center for General Welfare).**Results are found in Table 2.

In May and June 2023, we contacted the social services of all Flemish psychiatric hospitals as well as all psychiatric wards in general hospitals, by telephone to request their participation in this study. The names and telephone numbers of the psychiatric hospitals and the general hospitals with psychiatric wards were found on the website of the Flemish government (20). After carefully explaining the intent of the survey on behalf of the task force group “poor makes sick, sick makes poor” of the EGMH, we asked for oral informed consent, emphasizing that the participation was totally voluntarily and anonymous. After explicit oral informed consent, the “homelessness-in-mental-health-questionnaire” was then sent by mail to the social service worker, with the request to return it completed after 6 weeks at the latest. We estimated the task time to a maximum of 30 min. The descriptive statistical analysis was limited to calculating averages (arithmetic means) and medians of the percentages in the questionnaires (questions 1–5, 8, and 10).

## 3 Results

The questionnaire was answered a total of 24 times by employees of the social service of the respective psychiatric facilities: 11 responses out of the 40 psychiatric wards in general hospitals and 13 out of 30 psychiatric hospitals.

### 3.1 Quantitative results

In the quantitative part of the questionnaire, the total number of homeless patients in the inpatient setting on an annual basis was estimated to an average 19.5% of the total inpatient population, with a median of 13.8%, and a maximum of 70%. On the percentage of homeless patients who remain in admission longer than strictly necessary purely because of the lack of housing, estimates were made between 0 and 90%, with a mean of 18% of and a median of 5%. On average 13.7% homeless psychiatric patients (median 2%) in an inpatient setting are referred to a residential care facility such as an assisted living facility for the older adults or a psychiatric nursing home, despite the fact that they are able to live independently if sufficient suitable housing would be available.

In some cities, a very limited number of homes from the social housing corporation are reserved for psychiatric patients without requiring them to go through the regular waiting list. These accelerated referrals from psychiatric facilities to social housing companies are dismally low, averaging about 6.5% for homeless psychiatric patients. Almost all homeless patients (average 78.2%, median 100%) who are assigned housing through an accelerated referral system to social housing are required to accept residential counseling from before moving in until several years after. Exactly half of participating facilities reported having a protocol on how to deal with homeless psychiatric patients. Only one facility had no active policy to prevent eviction of hospitalized psychiatric patients. All others did have one, and this includes organizing household help or initiating a guardianship. More than 80% of the participating psychiatric facilities take active steps when a patient in a precarious living situation is threatened with eviction. They try to stop it by contacting the landlord or seeking legal help.

Social service respondents reported spending an average of 27.4% (median of 20%) of their work time on housing issues and homelessness. The results of this part of the quantitative questions are found in Table 2.

**Table 2:** Results of the quantitative part of the questionnaire.

	Average	Median	Range
Percentage homeless patients per year	19,5%	13,8%	3–70%
Percentage homeless patients with prolonged stay in hospital per year	18%	5%	0–90%
Unnecessary referral to residential care facility	13,7%	2%	0–80%
Accelerated referral to social housing	6,5%	0%	0–60%
Compulsory support in social housing	78,2%	100%	0–100%
Protocol homelessness	52,2%	100%	0–100%
Protocol prevention eviction	95,8%	100%	0–100%
Implementation protocol prevention eviction	85,9%	100%	0–100%
Percentage working time on housing problems	27,4%	20%	5–65%

Other quantitative results were the following: the respondents give an average rating of 6.8 out of 10 for the appropriateness of homes provided by social housing associations to homeless patients with mental health problems. For respondents, a landlord of an independent property on the open market has poor insight into the housing needs of a person with mental vulnerability, with a rating of only 2.9 out of 10. Individuals involved in policymaking, politics, or housing associations score slightly better, but still fall significantly short with an average rating of 3.8 out of 10.

### 3.2 Qualitative results

In the qualitative part of the questionnaire, all respondents indicate that there are collaborative efforts with social housing corporations, social service organizations, temporary homeless shelters, and with cities and municipalities. Two institutions also have contacts with hotels and monasteries. At the same time, all participants emphasize that there are hardly any priority measures for homeless psychiatric patients. On the contrary, psychiatric vulnerability seems to act as a barrier to potential referrals rather than opening doors. Social support organizations even look insistently towards psychiatric facilities to extend the duration of hospitalization as long as a housing solution is not available.

The most significant concern highlighted by all respondents is a shortage of adapted housing capacity. First and foremost, they are convinced that there should be a well-established network of crisis residences where homeless psychiatric patients can immediately find shelter, without it necessarily being a hospital bed and without their life on the streets escalating into a severe psychiatric crisis. For the respondents, there is a need for increased investment in infrastructure and manpower to address housing issues effectively. It is important to consider the preferences of the patients themselves, who often find communal living arrangements not feasible and prefer to have a place of their own. According to 13 respondents, a peaceful neighborhood is the most essential for a suitable residence for patients with mental vulnerability. 7 respondents mention the importance of sufficient nearby physical and mental healthcare, 4 of them a safe environment without nuisance and aggression. 8 respondents think a proper infrastructure for heating and electricity, sufficient comfort, and the opportunity for a cozy interior arrangement are essential features.

Additionally, there is a conviction regarding a structural problem in social housing. There is a shortage of housing units, and those available are often outdated. For individuals with mental vulnerabilities, it would be beneficial to have more support in processing and monitoring applications for social housing, especially for homeless individuals or those with psychiatric issues. This responsibility could be assigned to the social services of the city or municipality or to housing associations.

Another point of concern mentioned in the survey is the absence of suitable housing options for psychiatric patients who are not able to live entirely independently but also do not require a protected environment such as a psychiatric care facility or a residential care center for the older adults. Currently, the options seem to be either intensive support in a hospital, a protected environment, or living independently, with no middle ground. The seemingly endless waiting lists for suitable housing options lead people with mental health issues to lose hope, trapping them in a vicious cycle of illness. Searching for housing in the regular job market is highly susceptible to stigma and discrimination. This, too, contributes to a worsening of mental health problems.

## 4 Discussion

Almost 1 in 5 patients with a psychiatric illness in a psychiatric hospital or psychiatric ward in a general hospital in Flanders, Belgium, is homeless. 18% of all admitted patients stay longer in the hospital solely due to housing problems. This fact determines a significant part of the workload of social services associated with these admission settings. 27.4% of their working time is spent on solving these housing problems.

Searching for housing for a homeless patient during a psychiatric hospitalization is a challenging and difficult issue (21). A study in Canada revealed the need for adapted independent living options for psychiatric patients, but also more nursing homes and the increased specialization of existing residential resources (21). Homeless patients need shelter, and it is the duty of every facility to at least try to help find it when the patient is in admission. On the other hand, we must dare to question how much capacity of psychiatric beds in hospitals may be occupied by patients who are ready for discharge but do not have housing. This situation, indeed, contributes to longer waiting lists and hinders the patient flow from admission to discharge (21). Another study revealed that all homeless patients with prolonged stay in an acute hospital were relocated to a long-term care institution because the complexity of these patients’ needs increased the difficulty in finding appropriate resources in a timely manner (22). It is also known that the lack of social support at discharge and the absence of availability of housing solutions are predictors of psychiatric readmission (23).

This study demonstrates for the first time the intensive time investment of social therapeutic staff in housing issues of admitted psychiatric patients. More than a quarter of all available time for social workers in psychiatric institutions is spent addressing housing problems for patients, exceeding the budgeted workload. It is also frustrating work due to the limited prospect of resolving these issues satisfactorily due to the severe shortage of suitable housing options. In Flanders, there is no additional hospital staff is funded by the government to support the problem of homelessness among patients in hospitalization. Addressing the housing issues of psychiatric patients requires a coordinated effort from various parties, including the government, local authorities, housing associations, healthcare institutions, and other societal organizations.

The limitations of this study were the questionnaire, which included estimates of the number of homeless patients, the quality of housing, and insight into psychiatric problems, but lacked objective, reproducible measurements. Additionally, there was a high likelihood of participation bias from respondents who are frequently confronted with homelessness in their work, compared to non-respondents.

The absence of sufficient adapted housing options for patients with psychiatric issues, the complete lack of social housing for vulnerable groups, the shortage of suitable housing in general, and a lack of interest from politicians and policymakers in this crucial problem leads to increased general and mental health problems for this target group. This is an individual medical-ethical but also societal problem, due to increased total costs. An editorial in Lancet states that policy makers must make access to adequate housing a key social determinant of health and see housing as a core public health intervention (24). It also endorses that health-care professionals have a pivotal role to play (24). It is therefore essential to formulate an appropriate approach to change. We suggest 10 point-action plan for policy makers:

1. Ensure an adequate supply of social housing as the primary form of prevention. The most vulnerable group of psychiatric patients always runs the risk of homelessness without subsidized housing.
2. Provide sufficient accessible mental health care for everyone, to prevent individuals with severe mental health issues from becoming homeless due to their illness.
3. Offer sufficient alternative housing options for psychiatric patients who can no longer live independently but do not require hospitalization or other care facilities. Consider options such as sheltered housing or group living.
4. Establish crisis accommodation facilities where homeless psychiatric patients can temporarily stay while awaiting a permanent housing solution.
5. Introduce housing coaches in mental health care institutions, both residential and outpatient, with the sole responsibility of assisting patients in finding housing and mediating housing issues. This would relieve the regular social services from dealing with housing matters.
6. Integrate mental health into all housing-related policy initiatives, emphasizing the importance of sufficient green spaces around the homes of vulnerable patients, accessible recreational activities, and good connectivity with public transportation.
7. Mandate safe and sustainable energy provisions in housing for psychiatric patients, similar to connections for phone, television, and internet, and integrate them appropriately into the overall rental costs.
8. Appoint a national and regional coordinator responsible for housing policy for psychiatric patients, who is accountable to the government, similar to a flu commissioner or a representative for drug policy.
9. Address disturbances therapeutically through outreach teams or other forms of guidance. Never deprive individuals of their right to dignified housing.
10. Invest in structural monitoring of the combination of homelessness and mental health problems and set concrete goals to reduce homelessness.

Adequate housing was recognized as part of the right to an adequate standard of living in article 25 of the 1948 Universal Declaration of Human Rights of the United Nations (25). There is no doubt that this article is being violated many times, every day, also in wealthy developed countries, and especially for the most vulnerable people. It is the moral duty of mental health professionals to strongly protest against this and do everything in their power to combat this injustice.

## Data availability statement

The raw data supporting the conclusions of this article will be made available by the authors, without undue reservation.

## Author contributions

KC: Conceptualization, Writing – original draft. KB: Conceptualization, Data curation, Methodology, Writing – review & editing. MatH: Data curation, Investigation, Writing – review & editing. LS: Writing – review & editing. YW: Conceptualization, Writing – review & editing. GI: Conceptualization, Writing – review & editing. MarH: Writing – review & editing.
